# Recommendations for Nutritional Supplementation in Pediatric Oncology: A Compilation of the Facts

**DOI:** 10.3390/nu15143239

**Published:** 2023-07-21

**Authors:** Alexandra Podpeskar, Roman Crazzolara, Gabriele Kropshofer, Benjamin Hetzer, Evelyn Rabensteiner, Bernhard Meister, Petra Obexer, Christina Salvador

**Affiliations:** 1 Department of Pediatrics I, Division of Hematology and Oncology, Medical University of Innsbruck, Anichstr. 35, 6020 Innsbruck, Austria; 2 Department of Pediatrics II, Institute of Experimental Neonatology, Medical University of Innsbruck, 6020 Innsbruck, Austria

**Keywords:** children, nutrition, micronutrients, oncology, prevention, supplementation

## Abstract

Background: As one of the few modifiable risk factors, the importance of dietary patterns for both disease prevention and treatment outcome in pediatric oncology has gained increasing popularity. Malnutrition is associated with lower survival rates, tolerance to treatment, and quality of life. Yet, especially in children with malignancies, nutritional deterioration is common, and pediatric cancer patients often present with inadequate intake of micro- and macronutrients alike. Despite the reported widespread use of dietary supplements, few empirical data provide a basis for clinical recommendations, and evidence for their efficacy is inconsistent. Current literature lacks a systematic approach as to how and which supplements should be recommended for specific patients, types of cancer, or during specific treatments. The aim of this review is to highlight the role of the most frequently used nutrients in pediatric malignant diseases and to give a practical guide based on current scientific evidence. Methods: A comprehensive literature search was conducted on PubMed through April 2023 to select meta-analyses, systematic reviews, observational studies, and individual randomized controlled trials (RCTs) of macro- and micronutrient supplementation in pediatric oncology. The search strategy included the following medical subject headings (MeSH) and keywords: “childhood cancer”, “pediatric oncology”, “nutritional status”, “dietary supplements”, “vitamins”, “micronutrients”, “calcium”, “magnesium”, “vitamin D”, “zinc” “glutamine”, “selen”, and “omega-3 fatty acids”. The reference lists of all relevant articles were screened to include potentially pertinent studies. Results: The present review provides a comprehensive and updated overview of the latest evidence about the role of nutrition and diet in pediatric oncology, also focusing on different nutritional interventions available for the management of the disease. We summarize evidence about the importance of adequate nutrition in childhood cancer and the role of several micronutrients and critically interpret the findings. Possible effects and benefits of supplementation during chemotherapy are discussed, as are strategies for primary and secondary prevention. Conclusions: We here describe the obvious benefits of dietary supplementation for childhood cancer. Further large-scale clinical trials are required to verify the impacts of deficiencies and the possible benefits of supplementation and optimal dosages. (337 words).

## 1. Introduction

Childhood is a vulnerable period of rapid physical and cognitive development, during which nutrition plays a vital role in maintaining growth and overall health. Early diagnosis, prevention, and management of malnutrition are paramount as they affect the function of every organ system. Micronutrient inadequacy during childhood and adolescence can further exacerbate morbidity and mortality rates and create a high risk of chronic disease in adulthood. In children with malignancies, adequate dietary intake is even more important, as malnutrition negatively affects the effectiveness and tolerance of treatment, the patient’s Quality of Life (QoL), and can have many long-term health consequences [1,2,3].

## 2. Malnutrition in Pediatric Cancer

Children with cancer are regularly faced with nutritional challenges; it is estimated that up to 70% of pediatric patients with malignancies experience malnutrition [1,2,3,4]. The pathogenesis of malnutrition, which is defined as an imbalance in nutrient requirements and intake that causes adverse effects on growth, neurocognitive function, and body function, is complex. Malnutrition refers to both under- and overnutrition and includes deviations in micro- and macronutrient intake [5]. Current data suggest that, especially in children with solid tumors and in patients considered high-risk, undernourishment occurs more frequently [6]. At the same time, it has been realized that survivors of common pediatric malignancies are at risk for adult-onset diseases such as obesity, cardiovascular disease, and endocrine diseases [7]. Dyslipidemia, glucose intolerance, high blood pressure, and an increase in BMI can also be acute side effects of cancer treatments [8]. 

Even though the significance of malnutrition has been recognized in the past few years, no consensus exists regarding energy requirements in pediatric cancer patients, and even less is known about possible altered micronutrient needs.

## 3. Macronutrients and Energy Requirements

There are still considerable gaps in knowledge regarding whether and how energy needs differ in children with malignancies. In general, it has been estimated that energy needs are elevated by an increased metabolic rate due to tumor activity. However, the evidence for elevated energy needs in childhood cancer patients is inconclusive. Since cancer patients are less active than healthy children, increased requirements could be compensated by decreased needs for physical activity. A temporary stagnation in growth and alterations in body composition with a decrease in metabolically active tissue (fat-free mass) in children with cancer also seem to play a role [9,10,11,12,13].

The current recommendations suggest maintaining a diet corresponding to that of children of the same age and sex [14]. With regard to macronutrients, the optimal distribution of energy delivered by carbohydrates and lipids and the optimal ratio of energy to protein also appear to correspond to those of age-matched healthy children. 

During critical illness, the importance of proteins has been emphasized and considered necessary to compensate for increased degradation of endogenous proteins in response to stress hormones. High protein delivery could indeed be associated with benefits in the early phase of critical illness in adult Intensive Care Unit (ICU) patients. In conclusion, evidence concerning increased protein needs in children is scarce, and recommendations for adult patients must be individualized as the potentially harmful effects of higher protein provision in patients with acute kidney injury should be remembered [10,15,16,17,18,19]. “Alternative” or restrictive diets, such as the so-called neutropenic diet or the ketogenic diet, have little scientific support and are a potentially harmful strategy [20,21,22]. In addition to alterations in total energy balance, micronutrient deficiencies such as magnesium (Mg), zinc (Zn), selenium (Se), vitamin D (VD), and vitamin B12 (VitB12) were reported in pediatric cancer patients [23,24,25].

## 4. Micronutrients

Not only are micronutrient deficiencies naturally more common in children with severe acute malnutrition, but they are also frequently reported in children with cancer who are unable to meet their Recommended Daily Intake (RDI) needs. Micronutrient malnutrition can be masked by a patient’s phenotypical nutritional status, putting normally nourished children at risk for deficiency.

Micronutrients embrace vitamins, minerals, and trace elements, and, overall, deficiencies are reported in up to 96% of pediatric cancer patients. While observational data demonstrate an association between micronutrient deficiencies and adverse effects, studies regarding the prevalence, causes, and impact on outcomes are still scarce [26,27,28]. Apart from glutamine (Gln), VD, VitB6, and selen (Se), for which benefits could be documented in a small number of clinical trials (Gln *n* = 6, VD *n* = 3, VitB6 *n* = 1, Se *n* = 1), evidence of micronutrient supplementation is based exclusively on observational studies.

In the following, we will briefly summarize the most frequently reported deficient micronutrients and the most probable beneficial ones to supplement in pediatric patients with malignancies by discussing evidence from the literature and focusing on existing data from pediatric oncology. An overview is provided in Table 1.

### 4.1. Vitamin D

Vitamin D deficiency (VDD) is known as the most common nutritional deficiency and occurs mainly due to decreased sunlight exposure, inadequate dietary intake, malabsorption, or liver and renal diseases. Therefore, children with malignancies are theoretically more susceptible to VDD, and some studies have shown a higher prevalence of VDD in children with hematologic malignancies than in healthy children [23,24,55].

The role of VD in health, disease, and cancer pathogenesis has been increasingly recognized. VD promotes calcium absorption in the gut and maintains adequate serum calcium and phosphate (Ph) concentrations. It is important for enabling bone mineralization and is needed for bone growth and remodeling. VDD is thus associated with poor bone health, which is generally known to be compromised in childhood cancer patients [20]. Furthermore, VD has positive effects on the immune system. It acts as a powerful suppressor of interferon-gamma (IFN-y)-stimulated macrophage activation and therefore stimulates phagocytosis through macrophages. VD also regulates the adaptive immune response by orchestrating T and B cells [56]. VDD has recently been linked to an increased inflammatory mucosal state and impaired mucosal tissue barriers in children with acute lymphoblastic leukemia (ALL), as more cases of methotrexate (MTX)-induced oral mucositis have been reported in these patients [23]. Finally, laboratory and animal studies suggest that VD might inhibit carcinogenesis and slow tumor progression by promoting cell differentiation and inhibiting metastasis. Clinical trials to investigate the role of VD in the primary prevention of cancer in the adult population have provided insufficient evidence. However, higher VD levels might reduce cancer mortality rates [57].

These positive effects on overall health and the known high prevalence of VDD have led to VD supplementation in children with cancer, regardless of VD status.

Guidelines on VD supplementation for the general population are also considered appropriate for children with cancer and imply an adequate dietary VD intake with a minimal daily requirement of 400 IU (200–1100 mg) [30]. It has been agreed that a serum 25(OH)-D level of <50 nmol/L (20 ng/mL) is indicative of VD sufficiency. Supplementation with the standard initial dose of 2.000 IU VD is then recommended. Because of the frequent inability of children with cancer to meet their nutritional needs, regular measurements and surveillance of 25 OHD levels from diagnosis through therapy and the first years of follow-up are recommended [30,58]. Since the added benefit of VD supplementation in children with normal VD levels has not been demonstrated, routine supplementation is not suggested. Even more so, one should consider possible health risks from excessive VD intake, which may result in toxic hypercalcemia and, in extreme cases, renal failure, calcification of soft tissues, cardiac arrhythmias, and even death [59].

Irrespective of the lack of evidence for an effect of VD supplementation on bone mineral density (BMD) or fractures in children with cancer, ensuring adequate VD status and thereby mitigating one of the modifiable risk factors for bone health in children with malignancies seems important [60]. As a decrease in VD levels during MTX therapy was associated with a higher chance of developing oral mucositis in children with ALL, regular monitoring and, if required, VD supplementation might present another low-stress form of supportive therapy.

### 4.2. Calcium

Another important nutrient for bone health that is typically diminished in undernourished children is calcium (Ca). Together with VDD, hypocalcemia increases the risk of osteopenia, which is one of the late adverse effects of oncological treatment itself. The interfering factors in the regulation of bone metabolism in patients with cancer are complex, including the direct impact of the disease and the toxic effects of the drugs used in chemotherapy, as well as treatment-induced endocrine deficits and reduced physical activity [24,30,58]. Hypocalcemia may be further worsened by the use of glucocorticosteroids or other nephrotoxic agents that can cause hypercalciuria. Studies of bone metabolism in pediatric patients have so far focused on children with ALL, where osteopenia has been diagnosed at diagnosis [24].

Especially in children with VDD and VD substitution therapy, contemporary supplementation of calcium is crucial as VD enhances intestinal absorption of Ca and Ph, and adequate remineralization can only be ensured with sufficient Ca availability [30,61]. If recommended amounts of dietary Ca are not met, especially if VD is supplemented as well, 500 mg Ca per day is suggested as the standard supplementation dose for children [30,60,61].

### 4.3. Magnesium

Magnesium (Mg) is a cofactor in many enzymatic reactions, including the synthesis of proteins, nucleic acids, and adenosine triphosphate (ATP). It plays key roles in muscle contraction and relaxation, cell membrane stabilization, heart rhythm, and vascular tone.

Hypomagnesemia usually develops due to decreased intestinal absorption and increased renal excretion of Mg. As gastrointestinal (GI) losses and poor nutrition are commonly experienced by pediatric patients with malignancies, and increased tubular excretion due to renal toxicity as a consequence of polypharmacy is frequent, these patients are prone to hypomagnesemia [62,63].

Regarding the GI system, damage to the intestinal mucosa caused by many chemotherapeutic agents, especially fluorouracil (FU) and irinotecan, as well as MTX, provokes the loss of epithelial cells, which results in secretory diarrhea. Treatment with FU may further lead to decreased expression of the enzyme lactase in the intestinal brush border, resulting in lactose intolerance and causing osmotic diarrhea, thereby exacerbating GI losses. Some chemotherapeutics, like once again irinotecan, are known to cause cholinergically mediated increased intestinal motility. In the setting of steatorrhea, Mg deficiency often develops as Mg ions build complexes with undigested free fatty acids. Hypomagnesemia can worsen in the setting of acute pancreatitis, which is frequently reported after administration of L-asparaginase, a chemotherapy used in the treatment protocols for pediatric ALL. The accompanying hypocalcemia-induced hyperparathyroidism and resulting hypophosphatemia may also lead to concurrent renal Mg loss [64,65,66,67,68].

Increased renal excretion of Mg is common in children with malignancies as chemotherapeutics and various antibiotic and antifungal therapies are nephrotoxic. In the case of cisplatin, magnesium losses can be severe and persist despite discontinuation of therapy. In adult patients, the incidence of cisplatin-induced hypomagnesemia is reported to be as high as 90% [69]. Sustained excretion rates also apply for amphotericin B; other nephrotoxic agents include loop and thiazide diuretics, aminoglycosides, and cyclosporine [70,71,72,73].

Routine laboratory assessments are commonly based on the measurement of serum Mg levels, which may not correlate with total body Mg stores as the blood serum contains only 0.3% of total body Mg, most of which is in the red blood cells (RBCs). Thus, low levels of serum Mg do not always correlate with the development of symptoms, which further depends on the severity of Mg deficiency and the rate of its decline. Consequently, Mg replacement can be challenging, with oral replacement strategies generally being more effective at slowly replacing body stores but intravenous replacement being more effective at treating severe cases of hypomagnesemia [66,69,74].

### 4.4. Vitamin B Complex

B vitamins (B1 thiamine, B2 riboflavin, B3 niacin, B5 pantothenic acid, B6 pyridoxine, B7 biotin, B9 folate, and B12 cobalamin) are essential water-soluble micronutrients and key intermediates for critical cellular functions. They all conduct important tasks within the nervous system as coenzymes, including neurotransmitter synthesis and myelin production. Deficiencies of certain B-group vitamins, such as B1, B6, and B12, are associated with nerve dysfunction and can lead to peripheral neuropathy. Thus, they have been found to play a role in chemotherapy-induced peripheral neuropathy (CIPN), but currently there are no conclusive protective or treatment options [37]. Cobalamin (VitB12), pyridoxine (VitB6), and folate (VitB9) seem to be the most important members of the B vitamin complex, and deficiency was found in ALL patients who showed anemia on maintenance therapy [75]. Therefore, we will briefly discuss the most important aspects of the latter.

### 4.5. Vitamin B12

Vitamin B12 (VitB12), or cobalamin, is required for the development, myelination, and function of the central nervous system as well as the formation of RBCs.

VitB12 is absorbed from food by the body in two steps. In the first step, hydrochloric acid in the stomach and enzymes in the saliva of the mouth separate VitB12 from the protein to which it is attached in the food. In the second step, VitB12 is attached to a protein called the intrinsic factor (IF) and is later absorbed in the terminal ileum. For these reasons, a lack of VitB12 is mainly a result of malabsorption, either due to decreased secretion of hydrochloric acid, atrophic gastritis, or mucosal injury in the small intestine [76].Especially for children with hematologic malignancies, the diagnosis of VitB12 deficiency should not rely on abnormal hemoglobin levels or abnormal erythrocyte indices. Indicators of VitB12 deficiency are serum methylmalonic acid (MMA) and total plasma homocysteine levels, which both rise quickly as VitB12 status declines. As those indicators are also of poor specificity, it is suggested that patients with MMA levels be checked to see if their serum VitB12 level is less than 150 pg/mL, which would confirm the diagnosis of deficiency [77,78,79].

Children with VitB12 deficiency have been associated with higher risk rates for neurotoxicity [78]. Neurological side effects of chemotherapy, especially in those regimens including vincristine (VCR) and/or methotrexate (MTX), are common. Interactions of VCR with azole antifungals, one of the most commonly used drugs for antifungal prophylaxis in childhood leukemia, may further exacerbate neurotoxicity; VitB12 might be a beneficial protective agent in these patients [80,81].

### 4.6. Vitamin B6

Vitamin B6 (VitB6) comprises a group of six water-soluble chemical compounds; the active form, which is pyridoxal phosphate (PLP), serves as a cofactor for about 160 reactions in the body and has been estimated to be essential for 4% of all enzyme activities in the human genome. VitB6 participates in the transformation of carbohydrates, lipids, amino acids, and nucleic acids and is required for the synthesis of neurotransmitters. Its potential relevance to carcinogenesis and tumor growth has been discussed, as have associations between maternal intake and a decreased risk for the development of childhood ALL [37,38,82,83,84].

Supplemental VitB6 has been approved by the FDA for the treatment of nausea and vomiting during pregnancy since the late 1990s. Recently, a study by Mousavi-Hasanzadeh et al. suggested that vitamin B6 be considered an appropriate alternative for treating chemotherapy-induced nausea/vomiting (CINV) in children with malignancy as well [83].

Regarding chemotherapy-induced peripheral neuropathy (CIPN), B vitamins, especially pyridoxine, have been found to play a role in CIPN prevention. Köker et al. studied the effectiveness of pyridoxine plus pyridostigmine for therapy of vincristine-induced peripheral neuropathy (VIPN) in children with acute lymphoblastic leukemia and considered it a possible treatment option [36,37].

Once again, it is important to maintain the correct balance of VitB6 because several reports have indicated neurotoxic effects when consumed in excess [84].

### 4.7. Vitamin B9

Because of its role in the synthesis of purine and pyrimidine and in the DNA repair mechanism, folate (VitB9) is especially important during phases of rapid cell growth. As a cofactor in many single-carbon transfer reactions, VitB9 participates in multiple important reactions such as DNA methylation, amino acid homeostasis, and redox defense [82,85,86]. Reduced folate levels are held accountable for chromosomal breaks due to the massive incorporation of uracil into human DNA. Its crucial role in maintaining genomic integrity is affirmed by many case-control studies that indicate a possible protective effect of maternal folate supplementation for childhood cancers such as ALL [85]. Regarding supplementation during pregnancy, folate intake is probably best known for its role in reducing the risk of birth defects of the brain and spine, as high concentrations are especially important during neurogenesis. Folate deficiency is further known to manifest as disordered hematopoiesis and bone marrow dysfunction [86].

However, folate metabolism is complex, and supplementation might also stimulate DNA methylation, potentially playing a role in carcinogenesis, depending on the dosage and the timing of the exposure.

After HD-MTX infusions, folinic acid is administered to reduce toxic side effects in most protocols for pediatric ALL, osteosarcoma, or Non-Hodgkin lymphoma. Additional administration should excepted therefrom be limited to folate-deficient patients or patients with a risk for malabsorption or poor diets [38,86,87].

### 4.8. Vitamin C

Vitamin C (VitC) is a water-soluble vitamin that is mainly promoted as an antioxidant supplement to support immune health. It is essential for the formation of collagen and wound healing and improves the absorption of non-heme iron and endothelium-dependent vasodilatation [20,88,89]. Multiple hypotheses about the anti-tumor effects of VitC mainly refer to its function as a pro-oxidant and the formation of reactive oxygen species (ROS) that exert direct cytotoxic activity on cancer cells. In adult studies, increased chemotherapy-associated adverse effects have been observed with inadequate VitC intake, and improvements in CINV following administration of intravenous VitC have been anecdotally reported. The antiemetic effect of VitC may be mediated through its antioxidative effects since CINV has been attributed to ROS-related gut injury [39,40].

VitC deficiency is rare, and supplementation is generally considered safe. However, adverse effects include diarrhea and the increased formation of kidney stones. The recommended minimum intake of VitC varies from 50 mg/day in infants to 110 mg/day in adolescents [90,91].

### 4.9. Zinc

Zinc (Zn) is an antioxidant trace element that is essential for tissue repair, carbohydrate tolerance, immune function, and the gastrointestinal, central nervous, skeletal, and reproductive organ systems. Zn is ubiquitous within cells and executes several catalytic, structural, and regulatory functions. It is a cofactor for DNA synthesis and important in cell proliferation [41,42,92].

Zn-dependent mechanisms have been extensively studied, and the effectiveness of Zn in the treatment of acute diarrhea in young children has been demonstrated in several studies [41]. As Zn may increase the gastrointestinal epithelial barrier function, supplementation seems to lead to a reduction in the incidence and severity of oral mucositis in children with ALL [42].

Zn intake is closely related to protein intake. Thus, children with cancer seem to be particularly at risk. The general requirements of malnourished children are estimated to be between 2 mg/kg and 4 mg/kg of body weight. These requirements are much higher than those for healthy children (0.17 mg/kg at 1–3 years), presumably because of prior Zn depletion and reduced Zn absorption due to changes in the intestinal tract [32].

### 4.10. Selenium

Se is an essential trace element that mainly exerts its effect via its incorporation into proteins as selenoproteins. Selenoproteins are enzymatically active and directly involved in redox reactions. The anti-inflammatory, antirheumatic, and antiviral effects of Se are being discussed [44].

A recent study by Viera et al. proposed a beneficial effect of supplementation with Se on nausea, fatigue, and physical, renal, and liver function in pediatric cancer patients [45]. Currently, there are no data available regarding the Se requirement for children and adolescents. However, since most estimates show Se intake to be sufficient, routine supplementation is not recommended [34].

### 4.11. Glutamine

Gln is the most abundant amino acid (AA) in the human body and is considered conditionally essential as its endogenous production may become insufficient in the case of critical illness or cancer. Gln plays a pivotal role in many metabolic pathways and is necessary for optimal growth and cell division. As an essential precursor for nucleotide synthesis, the role of Gln in the development of malignancies has been extensively studied in recent years. Yet, evidence concerning future therapeutic targets and the role of Gln in the tumor microenvironment is still scarce [93,94].

However, much is already known about the other beneficial effects of Gln in patients with cancer, mainly due to its impact on the immune system and the treatment of mucositis.

As an important energy source for both enterocytes and leukocytes, Gln is essential for the maintenance of mucosal cell integrity and gut barrier function. Gln exerts major roles in the regulation and activation of both the innate and adaptive immune systems. It is necessary for the differentiation of B lymphocytes and the production of inflammatory cytokines. In addition, metabolites of glutamine metabolism promote the differentiation of macrophages, which in turn require Gln for the generation of nitric oxide (NO) and phagocytosis [48,95].

Gln has been proposed to protect the intestinal mucosa from the impact of aggressive chemotherapy, alleviate the severity of oral mucositis (OM), and support recovery. There are multiple studies supporting the use of oral (PO) glutamine or parenteral Gln in various adult cancer populations [46,48,50]. Because exogenous glutamine that is administered enterally is immediately taken up by mucosal cells in the upper gastrointestinal tract or by enterocytes, PO administration is generally preferred [48]. However, the efficacy of Gln for chemotherapy-associated mucositis remains variable in children, with some studies reporting cases of severe mucositis even with PO Gln supplementation. The authors suspect decreased efficacy because of poor general compliance with oral medication during periods of high-dose chemotherapy [48]. Thus, Chang et al. investigated the effects of Gln-enriched parenteral nutrition on MTX-induced severe OM in children with ALL in 2017 and were able to show a significant reduction in the incidence and severity of OM in these patients [49]. However, there are also reports of higher relapse rates following hematopoietic stem cell transplantation (HSCT) in patients treated with parenteral Gln supplementation [96]. PO Gln was found to be effective in reducing the severity and duration of OM and the incidence of severe OM. A recent study by Widjaja et al. concluded that PO Gln (400 mg/kg/day) can prevent OM, shorten the length of treatment, and reduce the cost of care for pediatric ALL [51].

Supplementation with Gln has also been associated with positive impacts on the nervous system. Sands et al. proposed a possible protective effect of Gln on vincristine-induced peripheral neuropathy (VIPN). The authors reported improvements in sensory functions. However, no impact on motor vincristine-induced neuropathic signs and symptoms could be demonstrated [47].

For these reasons, children with ALL who are treated with high doses of methotrexate (MTX) and VCR are particularly affected by OM and/or VIPN and would therefore likely benefit from Gln supplementation.

### 4.12. Omega-3

Omega-3 (*n*-3) fatty acids are long-chain polyunsaturated fatty acids (PUFAs) and include α-linolenic acid (ALA), eicosapentaenoic acid (EPA), and docosahexaenoic acid (DHA). They are considered essential dietary nutrients and play essential roles in cell signaling and in the structure and fluidity of membranes. *n*-3 fatty acids are thought to have antitumor effects and have been used to prevent carcinogenesis and inhibit malignant cell growth in vitro and in vivo. Above all, *n*-3 PUFAs are known for their anti-inflammatory actions as they reduce the production of pro-inflammatory cytokines such as interleukin-1 β (IL-1β), tumor necrosis factor α (TNF-α), and interleukin-6 (IL-6). Benefits of *n*-3 supplementation also relate to complex metabolic improvements, in particular glycemic control and insulin sensitivity, and effects on blood lipid concentrations [52,53,97,98].

The use of *n*-3 PUFAs has been shown to minimize chemotherapy side effects and improve cancer-related malnutrition. For instance, Freitas et al. reported that a higher consumption of EPA resulted in less weight loss during chemotherapy [52], and Rogers BC et al. found a stabilization of the resting energy expenditure (REE) after EPA supplementation, with significantly increased quality of life and improved appetite [3].

The ability of *n*-3 PUFAs to integrate into cell membranes turns DHA, the most abundant FA in neural cells, into a promising supplement for neuroblastoma patients. DHA appears to augment the cytotoxicity of chemotherapeutic agents against tumor cells while protecting normal neural cells by reducing oxidative stress [99].

A recent paper by El Amrousy et al. reported the potential of *n*-3 fatty acids on doxorubicin-induced cardiotoxicity in children with ALL and concluded that supplementation might diminish early cardiac injury [54].

Administration of DHA and EPA appears to be a relatively non-toxic form of supportive therapy for children with malignancies. While evidence regarding proper dosage recommendations is scarce, the European Food Safety Guidelines of 100 mg/day (for children and infants up to 24 months) and ~250 mg DHA and EPA/day for adults seem appropriate [100,101].

Potential side-effects of *n*-3 PUFA are likely to be dose-dependent; potential risks include nausea and gastrointestinal adverse effects, diminished wound healing, and decreased platelet activity [53,102].

## 5. Adverse Events and Long-Term Consequences

Despite the indisputable importance of proper nutrition and micronutrient coverage, the question of whether dietary supplements can reduce treatment-adverse effects and improve survival remains largely unanswered.

It is common for parents of critically ill children to worry about their eating habits and resort to additional supplements in order to meet their child’s nutritional needs. Although the human body has efficient homeostatic mechanisms that regulate the absorption and retention of many nutrients, which decreases the likelihood of toxicities, the safety of long-term use of these supplements must not be overlooked [4,27,55].

Children tolerate the acute side effects of antineoplastic agents better than adults. However, a growing child is more susceptible to long-term consequences. Although dietary modification may provide successful treatment, it should be emphasized that any intervention might have a negative consequence on a child’s growth and development. Upper limits (UL) of nutrient intake should be monitored. There is a lack of international consensus on the actual ULs, and this is especially true for children, for whom ULs are commonly established through a downward weight-based extrapolation from adult ULs, which is not always appropriate. Hence, routine laboratory measurements are required [26,29,90,103].

## 6. Conclusions

We lack consistency and guidelines in the use of nutritional interventions in children. Therapies are limited to enteral, feeding tube supplements, or parenteral nutrition without consensus on the type, timing, and macronutrient composition of each treatment modality or on micronutrient supplementation. Malnutrition is an adverse prognostic factor in children with cancer, and its prevalence, especially regarding micronutrients, is highly variable. Complete nutritional assessments, including anthropometry, biochemistry, clinical, and dietary assessments, are recommended. Regular monitoring and follow-up of children with cancer during and after treatment is advised so that interventions can be implemented and evaluated. While their macronutrient intake fits the recommended proportions, the overall kilocalorie intake tends to be lower in children with cancer. However, regarding micronutrient intake, pediatric oncology patients seem to mirror their healthy peers. Therefore, until refuted, general dietary recommendations for children also apply to those affected by malignancies [1,2,4,10,14].

## Figures and Tables

**Table 1:** Micronutrient benefits and dietary reference values per day in pediatric oncology.

	Mechanisms of Action	Dietary Reference Values [29,30,31,32,33,34,35] (Per Day)	Special Areas of Interest in Pediatric Oncology
Vitamin D	- Immune function; - Bone health; - Carcinogenesis and tumor growth.	- 0–12 mo: 400 iu - 1–13 y: 600 iu - 14–18 y: 600 iu	- Higher incidence of om in patients with vdd [23,27]; - Primary prevention of osteoporosis.
B Complex Vit B12 Vit B6 Vit B9	- Versatile coenzymes for amino acid, carbohydrate, and fat metabolism; - Biosynthesis of neurotransmitters, myelination; - Immune function; - Hemoglobin production; - Carcinogenesis and tumor growth.	Vitb12 - 0–6 mo: 0.4 μg - 7 mo–1 y: 0.5 μg - 1–3 y: 0.9 μg - 4–8 y: 1.2 μg - 9–13 y: 1.8 μg - 14–18 y: 2.4 μg Vitb6 - 0–6 mo: 0.1 mg - 7 mo–1 y: 0.3 mg - 1–3 y: 0.5 mg - 4–8 y: 0.6 mg - 9–13 y: 1 mg - 14–18 y: 1.2 mg Vitb9 - 0–6 mo: 65 μg - 7 mo–1 y: 80 μg - 1–3 y: 150 μg - 4–8 y: 200 μg - 9–13 y: 300 μg - 14–18 y: 400 μg	Vitb12 - Deficiency associated with higher risk for cipn [36,37]. Vitb6 - Treatment for cinv, vipn [36,37]. Vitb9 - Maternal intake associated with risk for neuroblastoma [38].
Vitamin C	- Protein metabolism; - Biosynthesis of collagen, neurotransmitters; - Antioxidative properties; - Immune function; - Absorption of non-heme iron; - Carcinogenesis and tumor growth.	- 0–6 mo: 40 mg - 7 mo–1 y: 50 mg - 1–3 y: 15 mg - 4–8 y: 25 mg - 9–13 y: 45 mg - 14–18 y:75 mg	- Cinv [39]; - To support immune health [40].
Zinc	- Required for hundreds of enzymatic reactions (DNA synthesis, protein metabolism); - Immune function; - Wound healing, cell signaling.	- 0–6 mo: 2 mg - 7 mo–1 y: 3 mg - 1–3 y: 3 mg - 4–8 y: 5 mg - 9–13 y: 8 mg - 14–18 y: 9 mg 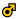 11 mg 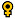	- Gastrointestinal epithelial barrier function [41,42,43]; - Diarrhea; - Reduction of OM in children with ALL.
Selenium	Selenoproteins - DNA synthesis; - Immune function; - Antioxidant; - Thyroid hormone metabolism, reproduction.	- 0–6 mo: 15 μg - 7 mo–1 y: 20 μg - 1–3 y: 20 μg - 4–8 y: 30 μg - 9–13 y: 40 μg - 14–18 y: 55 μg	- Benefits for nausea, fatigue, physical, renal, and liver function [44,45].
Glutamine	- Nucleotide substrate; - Regulation of genes related to metabolism, signal transduction, cell defense, and repair; - Immune function; - Antioxidant; - Protein metabolism.	0.3–0.5 g/kg/d	- Vipn [46,47]; - Reduction of om during mtx treatment [48,49,50,51].
Omega-3	- Immune function; - Antioxidant; - Carcinogenesis and tumor growth; - Glycemic control and insulin sensitivity.	Dha/epa - 0–24 mo: 100 mg - 2–18 y: 250 mg	- Malnutrition, ree ⇓ [52,53]; - Reduction of cardiotoxicity on doxorubicin [54].

## Data Availability

Publicly available literature was analyzed in this review. This data can be found on PubMed.

## Abbreviations

AA	amino acid
ALA	α-linolenic acid
ALL	acute lymphoblastic leukemia
ATP	adenosine triphosphate
BMD	bone mineral density
BMI	body mass index
Ca	calcium
CIPN	chemotherapy-induced peripheral neuropathy
CINV	chemotherapy-induced nausea/vomiting
CIPN	chemotherapy-induced peripheral neuropathy
DHA	docosahexaenoic acid
EPA	eicosapentaenoic acid
FA	fatty acid
FU	fluorouracil
GI	gastrointestinal
Gln	glutamine
HSCT	hematopoietic stem cell transplantation
ICU	Intensive Care Unit
IF	intrinsic factor
IFγ	interferon-gamma
IL-1β	interleukin-1β
IL-6	interleukin-6
MMA	methylmalonic acid
MTX	methotrexate
NO	nitric oxide
*n*-3	omega-3
OM	oral mucositis
Ph	phosphate
PLP	pyridoxal phosphate
PO	per os
PUFA	polyunsaturated fatty acid
RBC	red blood cells
RDI	recommended daily intake
REE	resting energy expenditure
ROS	reactive oxygen species
Se	selenium
TNF-α	tumor necrosis factor α
UL	upper limits
VCR	vincristine
VIPN	vincristine-induced peripheral neuropathy
VitD	vitamin D
VitB12	vitamin B12
VDD	vitamin D deficiency
QoL	quality of life
Zn	zinc
